# Symptom Presentation in Alzheimer's Disease and Its Impact on Caregiver Burden: A Qualitative Approach to Psychosocial Intervention Development

**DOI:** 10.1002/alz70858_101500

**Published:** 2025-12-25

**Authors:** K. Jayasankara Reddy V

**Affiliations:** ^1^ Christ University, Bangalore, Karanataka, India

## Abstract

**Background:**

The symptomatology of Alzheimer's disease varies across patients and stages of progression, influencing the intensity and nature of caregiver burden. While caregiving is often accompanied by physical and emotional challenges, the psychosocial aspects of caregiver experiences are often overlooked. This research aims to understand the psychosocial factors associated with caregiver burden, focusing on the influence of symptom presentation, and to develop a psychosocial intervention framework for caregivers.

**Method:**

To explore the psychosocial burden of caregivers of Alzheimer's patients and develop an intervention framework based on qualitative findings.

This qualitative study involved 13 in‐depth interviews with caregivers of Alzheimer's patients in India. Participants were selected across varying levels of patient symptoms as per the screening test, from early to advanced stages. Interview data were analyzed using a grounded theory approach to identify themes and sub‐themes related to the emotional, social, and neuro‐psychological aspects of caregiving.

**Result:**

Key findings included the identification of major psychosocial burdens, such as stress, guilt, anxiety, and fear for the future of the patient and family. Caregivers of patients in later stages reported higher levels of exhaustion, while those in earlier stages expressed confusion and lack of support. The research also uncovered gaps in current caregiver support programs, particularly in addressing the evolving needs as the disease progresses. The thematic model of psycho‐social rehabilitation will be presented based on the qualitative results. Additionally, a model for testing its efficacy will also be presented.

**Conclusion:**

This study emphasizes the need for dynamic psychosocial interventions that address specific caregiver challenges at each stage of Alzheimer's disease. Based on these insights, a structured psychosocial intervention model is proposed, focusing on emotional regulation, support networks, and adaptive coping strategies.

